# Assessment and validation of the TREAT-B score to assess the treatment eligibility of patients with chronic hepatitis B virus infection

**DOI:** 10.3389/fmed.2022.995857

**Published:** 2022-10-18

**Authors:** Kessarin Thanapirom, Sirinporn Suksawatamnuay, Panarat Thaimai, Sombat Treeprasertsuk, Piyawat Komolmit, Pisit Tangkijvanich

**Affiliations:** ^1^Division of Gastroenterology, Department of Medicine, Faculty of Medicine, Chulalongkorn University and King Chulalongkorn Memorial Hospital, Thai Red Cross Society, Bangkok, Thailand; ^2^Liver Fibrosis and Cirrhosis Research Unit, Chulalongkorn University, Bangkok, Thailand; ^3^Center of Excellence in Liver Diseases, King Chulalongkorn Memorial Hospital, Thai Red Cross Society, Bangkok, Thailand; ^4^Center of Excellence in Hepatitis and Liver Cancer, Department of Biochemistry, Faculty of Medicine, Chulalongkorn University, Bangkok, Thailand

**Keywords:** TREAT-B, chronic hepatitis B, treatment eligibility, treatment guidelines, antiviral

## Abstract

**Background and aims:**

Access to Hepatitis B virus (HBV) DNA testing to determine treatment eligibility is limited in low-income countries. Therefore, this study aimed to assess and validate the TREAT-B score proposed as the treatment threshold in an Asian cohort in determining the HBV treatment eligibility.

**Methods:**

A retrospective analysis was conducted on consecutive patients with treatment-naïve chronic HBV mono-infection who visited the liver clinic at Chulalongkorn University Hospital, Bangkok, Thailand, from 2016 to 2020. The 2018 American Association for the Study of Liver Diseases guideline was the reference standard.

**Results:**

Overall, 825 patients with chronic HBV infection were enrolled, comprising 409 (50.4%) males, with a median age of 50 (38–58) years. Of these, 216 (26.2%), 565 (68.5%), and 377 (45.7%) were eligible for treatment based on the AASLD, TREAT-B score, and simplified WHO criteria, respectively. The area under the receiver operating characteristics curve (AUROC) of the TREAT-B ≥ 2 was better than the simplified WHO criteria (0.69 vs. 0.62, *p* = 0.006) for selecting patients eligible for antiviral therapy. The sensitivity and specificity of the TREAT-B ≥ 2 were 96.3% and 41.4%, respectively. Applying the TREAT-B ≥ 3 improved the specificity (89.0%) and AUROC (0.80, 95% CI 0.76–0.84, but reduced the sensitivity (70.8%) for selecting eligible patients for HBV therapy.

**Conclusions:**

In resource-constrained countries where HBV DNA is unavailable, the TREAT-B score is an alternative criteria for indicating treatment eligibility. The TREAT-B score of ≥3 is highly accurate and may minimize the number of patients unnecessarily treated in Asian HBV patients.

## Introduction

Chronic hepatitis B virus (HBV) infection is a major global health problem affecting 296 million individuals and causing 820,000 deaths each year (1), mostly from liver cirrhosis and hepatocellular carcinoma (HCC) (1, 2). According to the World Health Organization (WHO) Global Hepatitis Report 2017, the prevalence of HBV infection is noted to be highest in the Western Pacific region (6.2%) and Africa (6.1%), which consist of a large number of Low and Middle Income Countries (LMICs) (3).

Toward eliminating HBV infection by 2030, improved treatment coverage for 80% of people eligible for antiviral therapy has been one of the WHO's interventional targets (4–6). Antiviral medications inhibit HBV replication and minimize the incidence of cirrhosis, HCC, and liver-related deaths (7). Several major international guidelines, such as the American Association for the Study of the Liver (AASLD) (8), the European Association for the Study of the Liver (EASL) (9), and the Asian Pacific Association for the Study of the Liver (APASL) (10), have recommended the criteria for initiating HBV treatment. However, most of these guidelines require tests rarely accessible and affordable in LMICs, including HBV DNA, liver elastography, and liver biopsy (11). Therefore, a simple score to indicate chronic HBV treatment eligibility is urgently needed to overcome barriers for treatment evaluation arising from the restricted availability of these tests.

The WHO guideline proposed simplified criteria specific for LMICs where HBV DNA is deemed inaccessible. These criteria consist of cirrhosis defined by clinical diagnosis or aspartate aminotransferase-to-platelet ratio index (APRI) of > 2 or persistently elevated alanine aminotransferase (ALT) on three ALT measurements for 6–12 months (12). However, the simplified WHO criteria is problematic since it requires several blood tests and medical visits before the treatment decision can be made. Recently, a new simple scoring system known as Treatment Eligibility for the Hepatitis B Virus (TREAT-B) has been developed and validated in Africa (13). This score is determined by the ALT level and hepatitis B e-antigen (HBeAg) serostatus. TREAT-B values ranged from 0 to 4. TREAT-B score of ≥2 had an accuracy with an area under the receiver operating characteristic curve (AUROC), sensitivity, and specificity of 0.85, 85%, and 77%, respectively, for selecting treatment-eligible patients with chronic HBV in African cohorts (13). The performance of TREAT-B for identification of HBV treatment eligibility has been evaluated in several subsequent studies in East and West Africa, Australia, Europe, and Vietnam (14–18). Several factors might affect the performance of TREAT-B, such as age (18), type of HBeAg assay (15), and the presence of HBeAg (19). However, the results were conflicting among the studies.

Southeast Asian countries are known to have a high HBV prevalence, with ~2% of the population chronically infected with HBV (3). Of these, ~5% were diagnosed, and only 1% of those eligible for treatment received antiviral therapy (20), limiting the WHO's target of increasing the treatment coverage to 80% by 2030. The prevalence varies among countries: 16.7% in the Philippines, 10.0% in Vietnam, 7.1% in Indonesia, and 4.0% in Thailand (20). The Thai Association for the Study of the Liver (THASL) published the practice guideline to manage chronic HBV in 2015 (21). However, this recommendation has similar limitations as in other international guidelines due to the requirement of special tests that are expensive and unavailable in most hospitals, particularly in primary and secondary hospitals. Therefore, this study aimed to assess and validate the ability of the TREAT-B score to identify treatment eligibility in an Asian cohort of patients with chronic HBV infection.

## Materials and methods

### Study participants

Consecutive patients aged ≥ 18 years with chronic HBV infection who visited the liver clinic at King Chulalongkorn Memorial Hospital, Bangkok, Thailand, from January 2016 to December 2020 were retrospectively enrolled in this study. The exclusion criteria were patients (1) with hepatitis C virus or human immunodeficiency virus coinfection; (2) with previous or current antiviral treatment for HBV; (3) who were pregnant; (4) with hepatocellular carcinoma; (5) with unreliable liver stiffness measurement using transient elastography (TE) (FibroScan^®^, Echosens, Paris, France), defined as an interquartile range (IQR)/median ratio of >30% (1); and (6) missing laboratory and virological data. The primary objective was to evaluate the performance of TREAT-B to identify patients who needed antiviral treatment using the international treatment criteria as a reference standard. Furthermore, the secondary objectives of this study were (1) to compare the accuracy of simple HBV DNA-free criteria, including TREAT-B and the Simplified WHO criteria, in selecting patients for antiviral therapy, and (2) to estimate the proportion of treatment-eligible patients based on various international recommendations.

Patients' demographic, clinical, and laboratory parameters were collected before initiating HBV therapy, including age, sex, serum aminotransferase, platelet count, HBeAg, and HBV DNA. HBeAg was determined using electrochemiluminescence immunoassays (Roche Diagnostics, Indianapolis, IN, USA). HBV DNA was quantified using real-time polymerase chain reaction COBAS AmpliPrep-COBAS TaqMan HBV test (Roche Molecular Systems, NJ, Branchburg, USA). Assessment of liver fibrosis was performed using non-invasive tests, including APRI and TE or liver histology. Advanced liver fibrosis (≥F3) was defined as TE of > 9 kPa. Cirrhosis was defined using radiological imaging, TE of >12 kPa, and/or clinical diagnosis. This study protocol was approved by the institutional review board, Faculty of Medicine, Chulalongkorn University (IRB 745/62). Furthermore, the study protocol adhered to the ethical principles of the Helsinki Declaration and followed the Good Clinical Practice guidelines.

### Hepatitis B treatment guidelines

Eligibility criteria for initiating HBV antiviral treatment recommended by several international guidelines were evaluated, including the AASLD 2018 (8), the EASL 2017 (9), APASL 2016 (10), THASL 2015 (21), Simplified World Health Organization (Simplified WHO) criteria (12), and TREAT-B score (13). Those of AASLD, EASL, APASL, and THASL primarily depend on HBV DNA, HBeAg, ALT level, and/or liver fibrosis staging by histopathology or elastography. The upper limits of the normal (ULN) for ALT are different among guidelines. In the EASL and APASL guidelines, the ULN for ALT normalization was defined as 40 IU/L, whereas in the AASLD and Simplified WHO, ULN for ALT was defined as 35 IU/L for men, 25 IU/L for women, 19 IU/L for men, and 30 IU/L for women, respectively. The cut-off value for determining ALT normalization was not mentioned in the THASL guideline. However, a previous study suggested that ALT of <34 and <32 IU/L for men and women were suitable for the robust ULN for serum ALT in Thai populations (22).

Two simple scores not requiring HBV DNA have been proposed for HBV therapy. The WHO recommendations provided simplified criteria consisting of cirrhosis (clinical diagnosis or APRI of > 2.0) or persistently ALT elevation of >3 visits during 6–12 months. Furthermore, a new simple TREAT-B score was developed and validated to identify HBV-infected patients in Africa (13). This score is calculated by adding the HBeAg serostatus (negative, 0 points; positive, 1 point) and ALT level (<20 U/L, 0 points; 20–39 U/L, 1 point; 40–79 U/L, 2 points; and ≥80 U/L, 3 points).

### Statistical analysis

Continuous variables were expressed as mean ± standard deviation or median and interquartile range. Categorical variables were expressed as numbers and percentages. The Fisher's exact test or Mann–Whitney *U*-test was used to compare continuous variables, whereas Pearson's chi-square or Fisher's exact test was used for categorical variables. Performance of the international guidelines, including sensitivity, specificity, positive and negative predictive value, the AUROC, and likelihood ratio for diagnosing HBV treatment eligibility, was evaluated. In general, an AUROC of 0.6–0.7 indicates satisfactory discrimination capacity to diagnose condition based on test, 0.7–0.8 is considered good, 0.8–0.9 is considered excellent, and more than 0.9 is considered outstanding (23). An AUROC comparison between two tests was made using Delong's et al. method (24) with MedCalc (MedCalc Software Ltd, Ostend, Belgium). The AASLD guideline was selected as the reference standard. Statistical analysis was performed using SPSS software (version 28, IBM Corporation, Armonk, NY, USA). A *p* < 0.05 was considered statistically significant.

## Results

### Patient baseline characteristics

**Figure 1:**
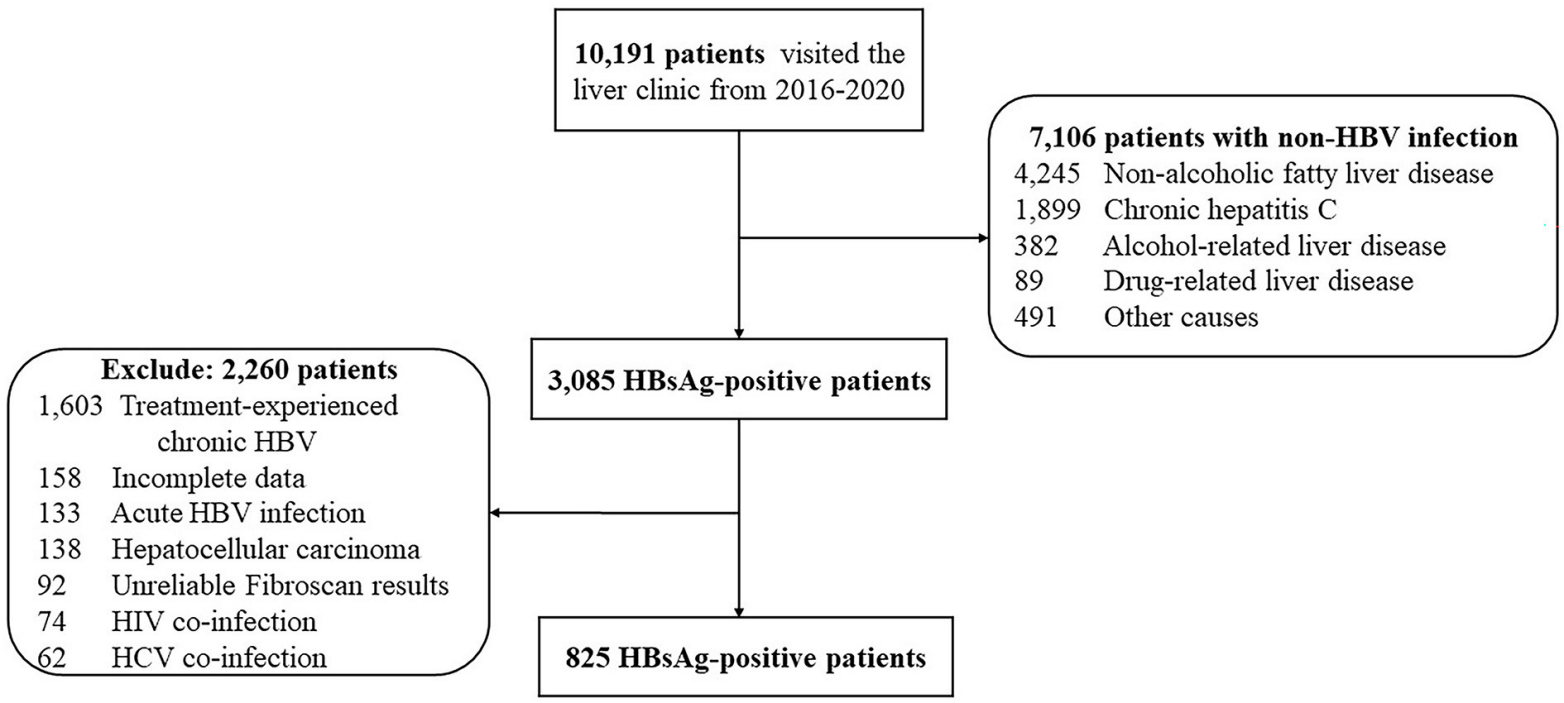
Flow chart of patient enrollment.

In total, 10,191 patients visited the liver clinic between January 1, 2016, and December 31, 2020. Among them, 3,085 patients tested for HBsAg; however, 2,260 patients were mostly excluded due to previous HBV treatment. Finally, 825 consecutive patients with untreated chronic HBV monoinfection were enrolled (Figure 1). Patients consisted of 409 men (50.4%) with a median age (interquartile range: IQR) of 50 (38–58) years. In total, 63 patients (7.6%) had liver cirrhosis, 154 (18.7%) were found to be positive for HBeAg and 305 (36.9%) had body mass index (BMI) ≥ 25 kilograms/meters squared (kg/m^2^). The median (IQR) alanine aminotransferase level, HBV DNA, and liver stiffness were 28 (19–48) IU/L, 3.3 (2.5–5.1) log IU/ml, and 5.4 (4.4–6.7) kPa, respectively (Table 1).

**Table 1:** Patient characteristics according to treatment eligibility.

	**Total (*n* = 825)**
Age, years	50 (38–58)
Male, *n* (%)	409 (50.4%)
Body mass index, kg/m^2^	23.7 (21.5–26.4)
HBeAg positive, *n* (%)	154 (18.7%)
Baseline AST, IU/L	24 (20–35)
Baseline ALT, IU/L	28 (19–48)
Platelet, 10^9^/L	235 (195–278)
HBV DNA, logIU/ml	3.3 (2.5–5.1)
Cirrhosis, *n* (%)	63 (7.6%)
Significant fibrosis, *n* (%)	205 (24.8%)
Liver stiffness, kilopascals	5.4 (4.4–6.7)
APRI	0.3 (0.2–0.4)

Data are shown as n (%) or median (quartile 1-quartile3). APRI, aminotransferase-to-platelet ratio index; kg/m^2^, kilograms/meters squared.

## Assessment of HBV treatment eligibility

**Figure 2:**
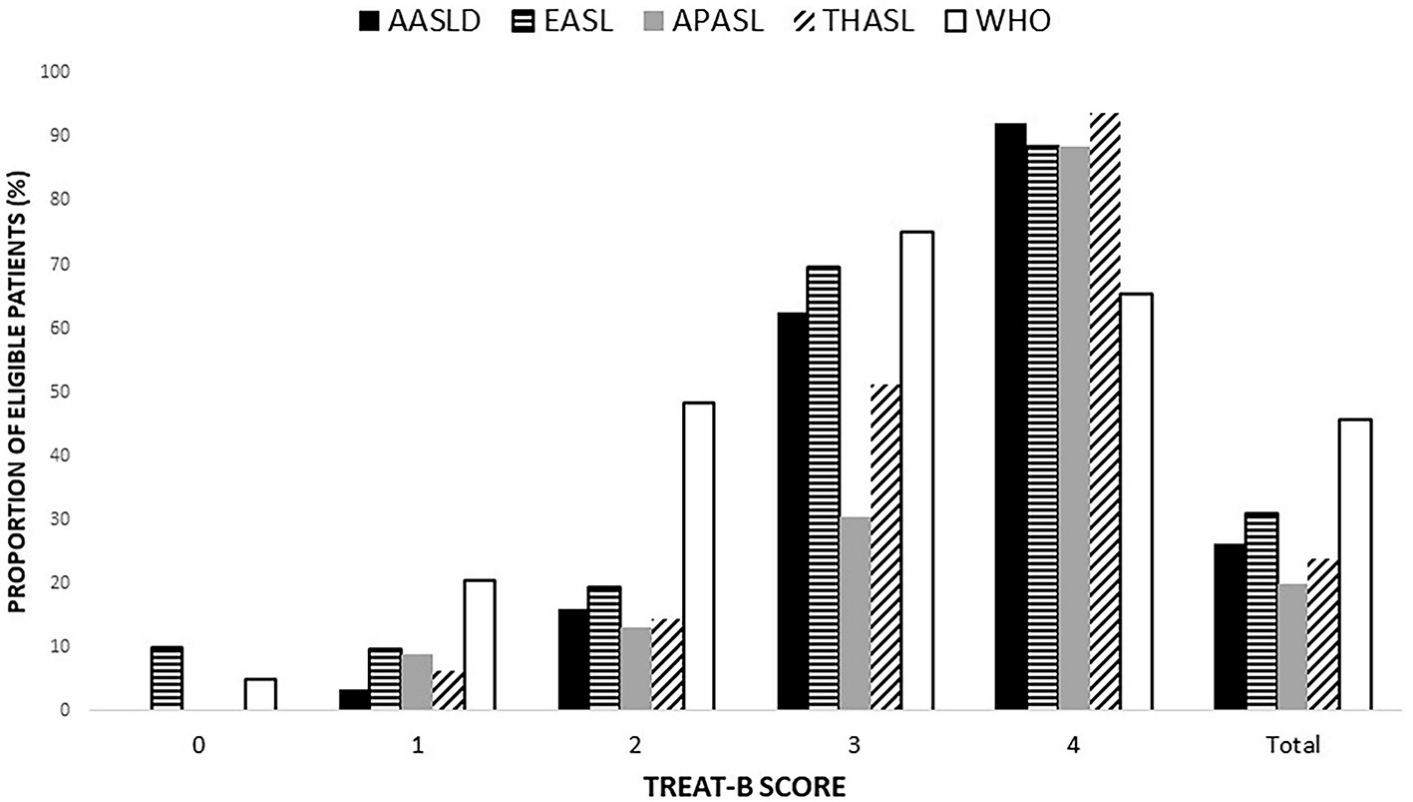
Proportion of patient eligible for treatment based on each of the international guidelines, expressed as a total point of TREAT-B (Data are shown in percentage). AASLD, the American association for the study of the liver; APASL, the Asian pacific association for the study of the liver; EASL, the European association for the study of the liver; THASL, the Thai association for the study of the liver; WHO, World Health Organization.

The proportion of eligible patients based on the international recommendations by the TREAT-B score is shown in Figure 2. The number of patients eligible for HBV treatment was 216 (26.2%), 255 (30.9%), 163 (19.8%), 196 (23.9%), 377 (45.7%), and 565 (68.5%) according to the AASLD, EASL, APASL, THASL, Simplified WHO, and TREAT-B of ≥2 criteria. The proportion meeting the international treatment criteria increased with a higher total TREAT-B score, except for the Simplified WHO criteria.

## Validation of the TREAT-B score compared to other international guidelines

Using the AASLD criteria as a reference standard, the TREAT-B of ≥2 [AUROC, 0.69; 95% confidence interval (CI), 0.65–0.73; *p* < 0.001] and the Simplified WHO criteria (AUROC, 0.62; 95% CI, 0.58–0.66, *p* < 0.001) had a satisfactory discrimination ability for HBV treatment eligibility. The AUROC of TREAT-B of ≥2 was higher than the Simplified WHO (0.69 vs. 0.62; 95% CI, 0.02–0.10; *p* = 0.006). In contrast to the criteria requiring HBV DNA, the APASL criteria (AUROC, 0.78; 95% CI, 0.74–0.82; *p* < 0.001), EASL (AUROC, 0.83; 95% CI, 0.80–0.87; *p* < 0.001), and THASL (AUROC, 0.87; 95% CI, 0.84–0.91; *p* < 0.001) guidelines exhibited an excellent discrimination accuracy (Table 2).

**Table 2:** Performance of the international guidelines to select patients eligible for antiviral therapy in reference to the AASLD 2018 guideline.

	**TREAT-B score**				**Simplified WHO**	**EASL**	**APASL**	**THASL**
**Cut-off**	**≥1**	**≥2**	**≥3**	**4**				
Accuracy	0.52	0.69	0.80	0.61	0.62	0.83	0.78	0.87
Sensitivity	100	96.3	70.8	22.2	71.6	80.6	62.6	79.0
Specificity	3.3	41.4	89.0	99.3	59.9	85.9	95.2	95.5
PPV	26.8	36.8	69.5	92.3	40.1	66.7	82.2	86.2
NPV	100	96.9	89.6	78.3	84.9	92.7	87.7	92.8
+ LR	1.0	1.6	6.4	31.7	1.8	5.7	13.0	17.6
– LR	0	0.1	0.3	0.8	0.5	0.2	0.4	0.2

APASL, the Asian Pacific Association for the Study of the Liver, EASL; the European Association for the Study of the Liver, PPV; positive predictive value, LR; likelihood ratio, NPV; negative predictive value, THASL; The Thai Association for the Study of the Liver, WHO; World Health Organization.

Regarding the cut-off TREAT-B criteria, a cut-off of 2 points had excellent sensitivity (96.3%) and NPV (96.9%), but low specificity (41.4%) and PPV (36.8%). Using the TREAT-B score of ≥3 improved the specificity (89.0%), PPV (69.5%), and AUROC (0.80; 95% CI, 0.76–0.84; *p* < 0.001), but reduced sensitivity (70.8%) and NPV (89.6%) for selecting patients for HBV therapy. The TREAT-B of ≥3 had a better accuracy when compared to TREAT-B of ≥2 (AUROC 0.80 vs. 0.69; 95% CI, 0.07–0.14; *p* < 0.001) and the Simplified WHO criteria (AUROC 0.80 vs. 0.62; 95% CI, 0.12–0.22; *p* < 0.001). Moreover, these findings remained consistent when changing the reference standard to the EASL, APASL, and THASL guidelines (Supplementary Table 1). TREAT-B of ≥3 (AUROC, 0.77, 0.71, and 0.77) had a good validity, whereas TREAT-B of ≥2 (AUROC, 0.66, 0.62, and 0.66) and Simplified WHO (AUROC, 0.63, 0.60, and 0.62) had a satisfactory capability for treatment eligibility indicated by EASL, APASL, and THASL guidelines (Supplementary Tables 1–3).

## Performance of TREAT-B in the subset of patients

The accuracy of TREAT-B of ≥2 (AUROC, 0.62–0.81) and TREAT-B of ≥3 (AUROC, 0.77–0.83) was deemed satisfactory when classified by age group (<40 vs. ≥40 years), the presence of HBeAg (negative vs. positive), and BMI (<25 vs. ≥25 kg/m^2^) (Table 3). As for the HBeAg status, 45.5% (*n* = 70/154) of HBeAg-positive patients met the AASLD treatment criteria, compared to 21.8% (*n* = 146/671) of HBeAg-negative patients. TREAT-B of ≥2 had comparable accuracy (AUROC 0.81 vs. 0.67) and sensitivity (95.9% vs. 97.9%) between HBeAg-positive and HBeAg-negative patients. However, the specificity was markedly decreased in HBeAg-negative (37.0%) than in HBeAg-positive (69.0%) patients. The sensitivity and specificity of TREAT-B of ≥3 were 55.7% and 97.6% in HBeAg-positive and 78.1% and 87.6% in HBeAg-negative patients.

**Table 3:** Performance of TREAT-B and Simplified WHO to select patients eligible for anti-HBV therapy in subgroup of patients.

	**Age**<**40 years (*****n*** = **231)**			**Age** ≥**40 years (*****n*** = **594)**		
	**TREAT-B ≥2**	**TREAT-B ≥3**	**Simplified WHO**	**TREAT-B ≥2**	**TREAT-B ≥3**	**Simplified WHO**
AUROC (95% CI)	0.74 (0.67–0.80)	0.80 (0.72–0.88)	0.62 (0.54–0.70)	0.67 (0.63–0.71)	0.80 (0.76–0.85)	0.62 (0.57–0.67)
*P*-value	< 0.001	< 0.001	0.009	< 0.001	< 0.001	< 0.001
Sensitivity	100%	71.4%	70.9%	94.8%	71.0%	71.5%
Specificity	47.3%	88.5%	60.8%	39.2%	89.1%	59.9%
PPV	39.2%	67.8%	39.4%	35.5%	69.6%	39.9%
NPV	100%	90.1%	85.3%	95.6%	89.7%	85.0%
	**HBeAg positive (*****n*** = **154)**			**HBeAg negative (*****n*** = **671)**		
AUROC (95% CI)	0.81 (0.74–0.88)	0.77 (0.69–0.85)	0.61 (0.52–0.70)	0.67 (0.63–0.72)	0.83 (0.79–0.87)	0.61 (0.56–0.66)
*P*-value	< 0.001	< 0.001	0.018	< 0.001	< 0.001	< 0.001
Sensitivity	92.9%	55.7%	79.7%	97.9%	78.1%	97.9%
Specificity	69.0%	97.6%	47.5%	37.0%	87.6%	36.0%
PPV	71.4%	95.1%	56.7%	30.2%	63.7%	34.3%
NPV	92.1%	72.6%	73.1%	98.5%	93.5%	86.7%
	**Body mass index**<**25 kg/m**^2^ **(*****n*** = **520)**			**Body mass index** ≥**25 kg/m**^2^ **(*****n*** = **305)**		
AUROC (95% CI)	0.69 (0.65–0.74)	0.77 (0.72–0.83)	0.59 (0.54–0.66)	0.62 (0.55–0.69)	0.81 (0.75–0.87)	0.61 (0.54–0.68)
*P*-value	< 0.001	< 0.001	0.002	0.003	< 0.001	0.004
Sensitivity	96.6%	63.8%	65.5%	96.2%	79.7%	79.2%
Specificity	42.4%	91.0%	58.6%	27.0%	82.6%	52.8%
PPV	37.7%	71.8%	37.0%	36.9%	67.0%	44.2%
NPV	97.1%	87.4%	82.0%	94.1%	90.2%	84.3%

AUROC, area under the receiver operating curve; PPV, positive predictive value; NPV, negative predictive value; 95% CI, 95% confidence interval.

Obese patients may have an overestimation of hepatitis and fibrosis, which affects the performance of the studied scores. Therefore, we further investigated how these scores performed in patients with and without overweight/obesity. A BMI of ≥25 kg/m^2^ was used to diagnose overweight/obesity based on the WHO recommendation (25). Overall, 26.5% (*n* = 138/520) and 30.7% (*n* = 94/305) of patients with BMI < 25 and ≥25 kg/m^2^ met the AASLD treatment criteria, respectively. The accuracy (AUROC 0.77 vs. 0.81), sensitivity of TREAT-B ≥ 3 (63.8% vs. 79.7%), and specificity (91% vs. 82.6%) were comparable between patients with and without overweight/obesity. Applying TREAT-B score of ≥3 substantially improved the specificity (82.6–91.0% vs. 27–42.4%) while decreasing the sensitivity (63.8-79.7% vs. 96.2-96.6%) compared to TREAT-B score of ≥2 in patients with BMI < 25 and ≥25 kg/m^2^ (Table 3).

Regarding advanced liver disease, 106 (12.8%) and 63 (7.6%) patients were diagnosed with stage ≥F3 liver fibrosis and cirrhosis, respectively. Of these, 83 patients (78.3%) with F3–F4 fibrosis and 52 with cirrhosis (82.5%) were determined eligible for treatment using TREAT-B of ≥2.

## Discussion

This current large cohort study assessed and validated the performance of a new simple TREAT-B score for determining the treatment eligibility of treatment-naïve patients with chronic HBV infection. Our main findings are as follows: (i) the number of patients eligible for HBV treatment was 26.2% and 68.5% identified with AASLD and TREAT-B of ≥2; (ii) the AUROC, sensitivity, and specificity of the TREAT-B score of ≥2 were 0.69 (95% CI: 0.65–0.73), 96.3% and 41.4%, respectively, when AASLD criteria were used as the reference standard; (iii) TREAT-B score had higher accuracy for discriminating patients who needed HBV therapy than the Simplified WHO criteria; (iv) in the Asian cohort of patients with chronic HBV infection, TREAT-B score of ≥2 did not perform well as described in the original article from the African cohort; (v) TREAT-B score of ≥3 improved the accuracy (AUROC, 0.80; 95% CI, 0.76–0.84) and specificity (89.0%), which might minimize the number of patients unnecessarily treated in the Asian HBV population. This finding remained unchanged even after using different international guidelines as the reference standard.

HBV infection is known to be prevalent in Thailand. The total estimated population in Thailand was 68.9 million in 2016 (data from the National Statistical Office of Thailand). Among them, ~2.8 million (4%) were chronically infected with HBV, and 898,000 individuals (32%) were found eligible for HBV therapy (20). HBV genotypes C (87.5%) and B (10.5%) are the major HBV genotypes in Thailand. The current study demonstrated that 26.2%, 30.9%, 19.8%, and 23.9% of HBV patients in Thailand required antiviral treatment classified by AASLD, EASL, APASL, and THASL guidelines. The APASL guideline has a lower potential for identifying HBV treatment eligibility than other international guidelines because it requires liver histology results in many circumstances. The Polaris study reported that only 11,900 Thai patients (1%) have received anti-HBV medications (20). One possible reason was that an assessment of HBV DNA and liver stiffness could only be performed at regional hospitals or tertiary care centers in Thailand. As a result, initiating HBV treatment in primary care centers is warranted. A study evaluating cost-effective methods involving a referral pathway for the management of patients with chronic HBV patients in primary care centers is still ongoing (26). Certainly, the simplified cascade of care for HBV is urgently needed, particularly in LMICs.

TREAT-B algorithm represents a simple and low-cost score, which is considered an alternative in selecting patients for chronic HBV treatment in remote areas, such as LMICs. The original article by Shimakawa et al. found that the TREAT-B threshold of ≥2 exhibited promising results with good sensitivity (85%), specificity (77%), and accuracy (AUROC, 0.85; 95% CI, 0.79–0.91) in multiple African countries (13). However, several subsequent validated studies showed contradictory results. The sensitivity and specificity of TREAT-B were reported as 53% and 83.4% in Ethiopia (16) and 69.8% and 70.4% in Burkina Faso (17), respectively. A multicenter study in Australia revealed that TREAT-B had greater sensitivity (91%) but lesser specificity (63%) in the hospital than in the community cohort (sensitivity, 70%; specificity, 88%) (18). TREAT-B of ≥2 demonstrated a sensitivity of 94.6% and specificity of 46.7% in a cohort of Vietnamese patients with chronic HBV (14), a finding consistent with this current study showing that using a cut-off of TREAT-B of ≥2 had excellent sensitivity (96.3%) but low specificity (41.4%) and fair accuracy (AUROC, 0.69; 95% CI, 0.58–0.66). Therefore, using the cut-off of ≥2, 59.6% of patients receiving antiviral would have been unnecessary. The possible explanations for why the results of subsequent validation studies were different from that of the original article might be the differences in HBV genotype and proportion of HBeAg-positive in enrolled patients among studies. HBV genotypes A, D, and E predominate in Africa, whereas genotype B predominates in Southeast Asia (27). The proportion of HBeAg-positive patients in Shimakawa's et al. study and our current study was 6% and 18.7%, respectively. Applying the TREAT-B score of ≥3 improved the specificity (87.8%) and AUROC (0.80; 95% CI, 0.76–0.84) but reduced the sensitivity (71.4%) for selecting patients for HBV therapy. Further cost-effective study of anti-HBV and TREAT-B is desperately needed. Despite the significant variation in sensitivity and specificity, all previous external validation studies consistently demonstrated that TREAT-B of **≥2** outperformed the Simplified WHO criteria in assessing patients' eligibility for antiviral therapy. The current study showed that TREAT-B score of ≥3 had a better accuracy (AUROC 0.80 vs. 0.62; 95% CI, 0.12–0.22; *p* < 0.001) and specificity (89% vs. 59.9%) when compared to the simplified WHO criteria. Even though both scores exhibited similar sensitivity (70.8% vs. 71.6%). Using a simplified score with high sensitivity but low specificity results in treating patients who don't require treatment. Although this approach makes it simpler to achieve the WHO's treatment goals, it will generate financial and therapeutic burdens on patients and healthcare systems and increases the risk of drug toxicity. Therefore, in areas where resources are severely limited, employing TREAT-B score of ≥3 might be appropriate for minimizing cases of unnecessarily prolonged treatment and these undesirable effects. The availability of resources in that country should be considered while selecting the proper score.

Regarding TREAT-B and subgroup of patients, the presence of HBeAg results in a higher TREAT-B score and a greater chance of commencing HBV treatment. Shimakawa et al. found that the AUROC of TREAT-B of ≥2 was similar between HBeAg-negative and HBeAg-positive (0.83 and 0.83) without affecting the sensitivity (13). This current study showed the accuracy was fair in HBeAg-negative patients (AUROC, 0.67) but excellent in HBeAg-positive patients (AUROC, 0.81). Moreover, the specificity of TREAT-B of ≥2 in HBeAg-negative patients (37%) was much lower than in HBeAg-positive ones (69%). On the other hand, TREAT-B of ≥3 increased the specificity in both HBeAg-negative (97.6%) and HBeAg-positive (87.6%) patients. Previous studies found inconsistent results about the influence of using point-of-care HBeAg testing on the performance of TREAT-B for determining HBV treatment eligibility. A Vietnamese cohort showed that TREAT-B was accurate when a rapid HBeAg test (SD Bioline, South Korea) was used (14). In contrast, a Malawian cohort found that three commercial HBeAg rapid diagnostic tests, including SD Bioline (Gauteng, South Africa), Creative Diagnostics (Shirley, NY, USA), and Biopanda Reagents (Belfast, UK) had insufficient sensitivity, potentially rejecting patients access to treatment based on TREAT-B score (15). The patient's age and BMI do not affect the TREAT-B's accuracy. The AUROCs of TREAT-B of ≥2 and ≥3 in patients aged < 40 years were 0.74 and 0.80, respectively, and 0.67 and 0.80 in those aged ≥ 40 years. Furthermore, The AUROCs of TREAT-B of≥2 and≥3 were 0.69 and 0.77 in non-overweight/obese patients and 0.62 and 0.81 in overweight/obese patients, respectively.

A previous study from an Australian cohort found that 42% of patients with advanced fibrosis or cirrhosis and 20% of patients with cirrhosis with treatment eligibility were missed using the TREAT-B algorithm (18). In contrast to our study, 21.7% of patients with ≥F3 fibrosis and 17.5% of patients with cirrhosis were missed in the TREAT-B of ≥2. The difference in enrolled patients might explain the discrepancy in results between the two studies. Howell et al. enrolled patients from a large hospital and several primary practice sites, whereas the current study enrolled patients from a large tertiary care hospital.

This study has some limitations. First, this study was conducted at the liver clinic in a tertiary care center. Thus, extrapolating our findings to patients with HBV in primary care hospitals may yield different results. The accuracy, positive, and negative predictive values are affected by the prevalence of the disease. In tertiary care centers, many patients are referred for initiating anti-HBV medication. Patients requiring treatment are more likely to be found in tertiary care hospitals than primary care hospitals. Therefore, the accuracy of TREAT-B when applied in primary care hospitals may differ from the results of the current study. Second, regarding the family history of HCC, the AASLD guideline (reference standard) stated that even though the patient has ALT < 2 of the upper limits normal and HBV DNA below thresholds, the HBV treatment should be considered if there is a positive family history of HCC. The current study discarded the family history of HCC in the international guideline due to only a few individuals had this information. Hence, applying the performance of TREAT-B score from our study to determine HBV treatment in this group of patients should be done with caution. Third, the data on alcohol drinking was missing in most patients due to the nature of retrospective study. This might impact the performance of the TREAT-B score, which relied on aminotransferase elevation.

In conclusion, TREAT-B is a simple and accurate alternative for determining patient eligibility for HBV treatment in Asia. TREAT-B score of ≥3 has high accuracy and may reduce the number of patients unnecessarily treated with lifelong medications. Utilizing TREAT-B may promote HBV elimination through improving the treatment program particularly in LMICs.

## Data availability statement

The original contributions presented in this study are included in the article/Supplementary material, further inquiries can be directed to the corresponding authors.

## Ethics statement

The studies involving human participants were reviewed and approved by the Institutional Review Board, Faculty of Medicine, Chulalongkorn University (IRB 745/62). Written informed consent for participation was not required for this study in accordance with the national legislation and the institutional requirements.

## Author contributions

KT and PTa conceived and designed this study. SS and PTh collected the data. KT analyzed the data and wrote original draft of the manuscript. KT, PK, ST, and PTa revised the manuscript. All authors read and approved the final manuscript.

## Funding

The study was supported by the Ratchadapiseksompotch Endowment Fund of Liver Fibrosis and Cirrhosis Research Unit (GRU 6105530009-1), the Ratchadapiseksompotch Endowment Fund (RA 65/021), Faculty of Medicine, Chulalongkorn University, the Thailand Science Research and Innovation Fund, Chulalongkorn University (HEA663000044), the Thai Red Cross Research Committee (2022), the Medical Council of Thailand (2021), the Royal College of Physicians of Thailand, and the Thai Association for the Study of Liver.

## Conflict of interest

The authors declare that the research was conducted in the absence of any commercial or financial relationships that could be construed as a potential conflict of interest.

## Publisher's note

All claims expressed in this article are solely those of the authors and do not necessarily represent those of their affiliated organizations, or those of the publisher, the editors and the reviewers. Any product that may be evaluated in this article, or claim that may be made by its manufacturer, is not guaranteed or endorsed by the publisher.
