# Supplementary material for: Assessment and validation of the TREAT-B score to assess the treatment eligibility of patients with chronic hepatitis B virus infection
Source: Front Med (Lausanne). 2022 Oct 18;9:995857. doi: 10.3389/fmed.2022.995857 (PMC9623013; doi:10.3389/fmed.2022.995857)
Supplement: Supplementary file 1 [file Table_1.DOCX]

**Supplementary Table 1: Performance of the TREAT-B and Simplified WHO score to select patients eligible for antiviral therapy in reference to the EASL 2017 guideline**

|  | **TREAT-B score** | | | | **Simplified WHO** |
| --- | --- | --- | --- | --- | --- |
| **Cut-off** | **>= 1** | **>= 2** | **>= 3** | **4** |  |
| Accuracy | 0.51 | 0.66 | 0.77 | 0.59 | 0.63 |
| Sensitivity | 99.2 | 90.2 | 63.9 | 18.0 | 73.8 |
| Specificity | 3.2 | 42.0 | 91.4 | 99.6 | 64.2 |
| PPV | 31.8 | 41.4 | 77.3 | 95.8 | 50.3 |
| NPV | 90.0 | 90.4 | 84.8 | 72.8 | 83.3 |
| +LR | 1.0 | 1.6 | 7.4 | 45.0 | 2.1 |
| -LR | 0.2 | 0.2 | 0.4 | 0.8 | 0.4 |

+LR, positive likelihood ratio; -LR, negative likelihood ratio; NPV, negative predictive value; PPV, positive predictive value; WHO, World Health Organization

**Supplementary Table 2: Performance of the TREAT-B and Simplified WHO score to select patients eligible for antiviral therapy in reference to the APASL 2016 guideline**

|  | **TREAT-B score** | | | | **Simplified WHO** |
| --- | --- | --- | --- | --- | --- |
| **Cut-off** | **>= 1** | **>= 2** | **>= 3** | **4** |  |
| Accuracy | 0.52 | 0.62 | 0.71 | 0.64 | 0.60 |
| Sensitivity | 100 | 87.1 | 59.5 | 28.2 | 69.6 |
| Specificity | 3.1 | 36.5 | 81.6 | 99.4 | 57.1 |
| PPV | 20.5 | 25.5 | 44.7 | 92.0 | 29.6 |
| NPV | 100 | 91.9 | 89.0 | 84.7 | 87.8 |
| +LR | 1.0 | 1.4 | 3.2 | 47.0 | 1.6 |
| -LR | 0.0 | 0.4 | 0.5 | 0.7 | 0.5 |

+LR, positive likelihood ratio; -LR, negative likelihood ratio; NPV, negative predictive value; PPV, positive predictive value; WHO, World Health Organization

**Supplementary Table 3: Performance of the TREAT-B and Simplified WHO score to select patients eligible for antiviral therapy in reference to the THASL 2015 guideline**

|  | **TREAT-B score** | | | | **Simplified WHO** |
| --- | --- | --- | --- | --- | --- |
| **Cut-off** | **>= 1** | **>= 2** | **>= 3** | **4** |  |
| Accuracy | 0.52 | 0.66 | 0.77 | 0.62 | 0.62 |
| Sensitivity | 100 | 92.3 | 66.8 | 23.5 | 73.1 |
| Specificity | 3.2 | 39.3 | 86.5 | 99.5 | 59.6 |
| PPV | 24.5 | 32.3 | 60.9 | 93.9 | 37.7 |
| NPV | 100 | 94.2 | 89.3 | 80.5 | 86.9 |
| +LR | 1.0 | 1.5 | 4.9 | 47.0 | 1.8 |
| -LR | 0.0 | 0.2 | 0.4 | 0.8 | 0.5 |

+LR, positive likelihood ratio; -LR, negative likelihood ratio; NPV, negative predictive value; PPV, positive predictive value; WHO, World Health Organization
